# A small unconditional non-financial incentive suggests an increase in survey response rates amongst older general practitioners (GPs): a randomised controlled trial study

**DOI:** 10.1186/1471-2296-14-108

**Published:** 2013-07-30

**Authors:** Sabrina Winona Pit, Vibeke Hansen, Dan Ewald

**Affiliations:** 1 University Centre for Rural Health, Sydney School of Public Health, The University of Sydney, 61 Uralba Street, PO Box 3074, Lismore, NSW 2480, Australia; 2 Clinical Advisor North Coast NSW Medicare Local, PO Box 957, Ballina, NSW 2478, Australia

**Keywords:** Surveys, Randomized controlled trial, Response rate, Family physician, Questionnaire, Incentives, Employment, Online

## Abstract

**Background:**

Few studies have investigated the effect of small unconditional non-monetary incentives on survey response rates amongst GPs or medical practitioners. This study assessed the effectiveness of offering a small unconditional non-financial incentive to increase survey response rates amongst general practitioners within a randomised controlled trial (RCT).

**Methods:**

An RCT was conducted within a general practice survey that investigated how to prolong working lives amongst ageing GPs in Australia. GPs (n = 125) were randomised to receive an attractive pen or no pen during their first invitation for participation in a survey. GPs could elect to complete the survey online or via mail. Two follow up reminders were sent without a pen to both groups. The main outcome measure was response rates.

**Results:**

The response rate for GPs who received a pen was higher in the intervention group (61.9%) compared to the control group (46.8%). This study did not find a statistically significant effect of a small unconditional non-financial incentive (in the form of a pen) on survey response rates amongst GPs (Odds ratio, 95% confidence interval: 1.85 (0.91 to 3.77). No GPs completed the online version.

**Conclusion:**

A small unconditional non-financial incentives, in the form of a pen, may improve response rates for GPs.

## Background

Low response rates in general practice research are common and lead to loss of power, selection bias ,unexpected budgetary constraints and time delays in research projects. Kellerman and colleagues [1] conducted a systematic review looking at how to increase response rates amongst physicians. Effective strategies were: monetary incentives, use of stamps on both outgoing and reply paid envelopes and shorter surveys. Non-effective strategies were: pre-notification, personalised mailouts and non-monetary incentives. VanGeest et al. [2] also performed a systematic review in 2007 amongst physicians and looked at two strategies amongst physicians: incentives and design based approaches. Contrary to expectations, small incentives of $1 were effective whereas non-monetary incentives were much less effective. Additionally, first-class stamps on reply paid envelopes, short surveys, personalised and studies endorsed by professional organisations were more effective. VanGeest et al. also found that postal and telephone strategies were more effective than fax and web based surveys, and mixed methods also appeared to be effective. In 2010, the US National Cancer Institute conducted a large-scale workshop to investigate how best to improve the quality of surveys of physicians and medical groups. One of the key issues discussed was that more research was required into a range of response incentives, including non-monetary incentives [3].

To further advance the current knowledge base, our study intended to test the effectiveness of an unconditional incentive to increase response rates amongst GPs. One study in the literature reported that the inclusion of an attractive pencil increased the response rate to a second mail-out amongst physicians [4]. Hence, this study examined the effectiveness of offering a small unconditional non-financial incentive on survey response rates amongst GPs. It was hypothesised that an unconditional non-financial incentive in the form of an attractive pen would lead to a higher response rate compared to using nothing.

## Methods

This study was part of a larger study investigating how to improve the sustainable employment of ageing GPs in rural Australia. The study incorporated both an anonymous survey and optional follow up interviews with participating GPs about sustainable employability. The survey questions pertained to socio-demographic and practice characteristics, retirement intentions, work ability, burnout, work satisfaction and health. The sub-study reported here was conducted to improve the response rates as part of the larger pilot-study.

The following strategies that have demonstrated an effect on physicians’ response rates in experimental studies were used to increase response rates for both groups: use of multiple stamps on reply paid envelopes [1,5], short survey [1,5], endorsement by a professional organisation [2], a topic relevant to the participants [5], multiple reminders [5], university based research [5], guaranteed confidentiality [5].

### Trial design

The study was a randomised controlled trial.

### Participants and setting

Eligible GPs were those who practiced in the Northern Rivers region of NSW, Australia, were aged 45 years and above, and included those doing any related GP work. Participants were drawn from a database held by the local GP Network which is routinely updated and includes all GPs in the region. In October 2011, all eligible participants were sent an invitation package consisting of a letter of support from the local GP Network, a study information letter, a three page quantitative survey, an on-line survey web-address, and an optional invitation to take part in an interview. Because the surveys were returned anonymously, completed surveys were taken as consent. Two reminders were sent two and four weeks after the initial invitation.

### Interventions

The intervention group was sent a non-conditional non-financial reward in the form of a nice looking pen, equating to a monetary value of $2, in the first mail-out. The pen had a mountain view with clouds, the University’s name, and a statement “Doctors working on” printed on it. No further incentives were sent. The control group did not receive the pen.

### Outcomes

The primary outcome measure was the response rate of returned surveys.

### Sample size

The GP Network’s database had 125 eligible members aged 45 years and over. Given the small sample, all eligible study participants were included in this sub-study. A similar study surveying early retirement amongst GPs who were recruited from a similar organisation in Australia, achieved a response rate of 59% [6]. Assuming a power of 80%, significance level of 5%, and a response rate of 60% in the intervention group and 35% in the control group, the estimated sample size required to detect a 25% difference was 122 or 61 in each group.

### Randomisation

A random number generator in SAS [7] was used to create random numbers for the two groups. No restriction was made. The list of GPs in the GP Network database were numbered consecutively and GPs were randomly allocated to the intervention or control group. In consultation with GP advisors and based on the literature [5], it was anticipated that higher response rates would be achieved if the survey was anonymous to allow GPs to take part and answer sensitive questions about their health. Therefore, the surveys did not include an identifying number but only one ‘letter’ to identify which group the returned surveys belonged to.

### Blinding

Participants were blinded because they were not made aware in advance that the trial was carried out. The sequence allocation was not concealed from the project manager or the GP Network staff. However, participants were unaware of treatment allocation.

### Statistical methods

The odds ratio for response rates was calculated for the first mail out, second reminder and last reminder. Chi-square analyses were used to compare categorical variables between groups. T-tests were used to compare continuous variables.

### Evaluating nonresponse bias

In line with Johnson and Wislar [8], nonresponse bias was examined by:

1) comparing the survey responses between early responders (initial mail-out) and late responders (2nd and 3rd reminders), under the assumption that later responders are most similar to non-responders; and

2) Comparing of survey sample with other data sources: the Australian Institute of Health and Welfare, National Health Workforce Data Set 2011 [9].

## Results

The total number of participants aged 45 years and over in the trial was 125. No GPs completed the survey online. Table 1 displays the number of participants randomised and the number responding based on first, second and third mail out. Invitations were sent between October to December 2011. The overall response rate was 54.4%. The final response rate for the intervention and control group were 61.9% and 46.8% respectively and was not statistically significant (Chi-square = 2.89, P = 0.089). The odds ratio for the final response rates for the intervention versus the control group was 1.85 (95 CI% 0.91, 3.77).

**Table 1:** Number of participants randomised and numbers responding based on first, second and third mail out (Number randomised = 125)

	**Total N**	**Total responders n (%)**	**Responders by group, n (%)**	**OR (95% CI)**
**Initial mail out**				
Pen	63		27 (42.9%)	1.46 (0.71- 3.02)
No pen	62		21 (33.9%)	1.00
*Total*	125	48 (38.4%)		
**First reminder**				
Pen	63		36 (57.1%)	1.85 (0.91 - 3.75)
No pen	62		26 (41.9%)	1.00
*Total*	125	62 (49.6%)		
**Final reminder**				
Pen	63		39 (61.9%)	1.85 (0.91 - 3.77)
No pen	62		29 (46.8%)	1.00
*Total*	125	68 (54.4%)		

Based on survey responses, there were no statistically significant differences between the intervention and control group in relation to socio-demographic, practice and perceived work issues (Table 2).

**Table 2:** Comparison of characteristics between responders between intervention (‘pen’) and control group (‘No pen’)

	**‘Pen’ n = 39**	**‘No pen’ n = 29**	**P**
	**%**	**%**	
Female	26%	36%	0.37^a^
Group practice (>1 GP)	84%	96%	0.22^a^
Reported fair or poor health	18%	31%	0.23^a^
	**Mean (sd)**	**Mean (sd)**	
Age (years)	57 (6.9)	54 (6.8)	0.12^b^
Years in general practice	27 (9.1)	24 (10.0)	0.36^b^
Job satisfaction scale (‘0’ not satisfied to ‘10’ extremely satisfied)	7.3 (2.2)	7.7 (1.9)	0.42^b^
Burnout scale (‘0’ not burnt out to ‘10’ extremely burnt out)	3.5 (3.1)	2.6 (1.9)	0.15^b^

^**a**^**χ**^**2**^ test, ^b^ t-test.

Evaluation of nonresponse bias demonstrated that there was no difference between early responders and late responders on survey responses (Table 3) and the distribution of gender and age was very similar compared to the rural general practice population on national level (Table 4).

**Table 3:** Evaluation of nonresponse bias: Comparison of characteristics between early responders (initial mail-out) and late responders (2nd and 3rd reminder)

	**‘Early’ n = 48**	**‘Late’ n = 29**	**P**
Female	25%	40%	0.24^a^
Group practice (>1 GP)	94%	79%	0.10^a^
Reported fair or poor health	23%	25%	0.89^a^
	**Mean (sd)**	**Mean (sd)**	
Age (years)	55 (6.5)	56 (8.1)	0.80^b^
Years in general practice	25 (9.4)	26 (9.8)	0.64^b^
Job satisfaction scale (‘0’ not satisfied to ‘10’ extremely satisfied)	7.4 (2.0)	7.6 (2.3)	0.77^b^
Burnout scale (‘0’ not burnt out to ‘10’ extremely burnt out)	3.3 (2.5)	2.8 3.0)	0.46^b^

^**a**^**χ**^**2**^ test, ^b^ t-test.

**Table 4:** Evaluation of Nonresponse bias: Comparison of survey sample with other data sources

	**This study**		General rural GP population*	
	**n**	% (95%CI)**	**n**	**%**
**Gender**				
Male	47	70% (59% to 81%)	3238	71%
Female	20	30% (19% to 41%)	1354	29%
**Age:**				
45-54	32	47% (35% to 59%)	2290	50%
55-64	28	41% (29% to 53%)	1660	36%
65+	8	12% (4% to 20%)	642	14%

* Source: Australian Institute of Health and Welfare, National Health Workforce Data Set 2011[9]. ** 95% confidence intervals.

## Discussion and conclusions

### Summary

The response rate for GPs who received a pen was higher in the intervention group (61.9%) compared to the control group (46.8%). The incentive group resulted in a 15.1% higher response rate compared to the control but this did not reach statistical significance.

### Strengths and limitations

A strength of this study is that, to our knowledge, this is the first study that investigated the use of a nice pen to improve response rates amongst GPs compared to a simple control group. Another strength is that a lot of the techniques that have been demonstrated to improve response rates were implemented in this study. Also, nonresponse bias analyses demonstrated there was little nonresponse bias and age and gender distribution was similar to the rural general practice population. The major limitation of the study is the small sample size, which led to the study being underpowered; Assuming a power of 80%, significance level of 5%, and given our response rate of 62% in the intervention group and 47% in the control group, the estimated sample size required to detect a 15% difference was 340 or 170 in each group. This needs to be taken into account when interpreting the results. It is likely that a larger sample size would have led to statistically significant results. The study formed part of a larger pilot study and the sample size was therefore confined to the current sample. Another limitation of the study is not being able to link survey data to responders. However, it was felt that identifiable information would have limited response rates and GPs ability to answer the questions truthfully. Another limitation of the study is that the results may only be generalizable to GPs.

### Comparison with existing literature

Edwards and colleagues [5] found that unconditional incentives were an effective strategy to increase postal survey response rates in the general population. The odds of responses also increased slightly when using non-monetary incentives. Non-monetary incentives also increased the odds of higher responses for electronic surveys. However, a systematic review found that the majority of physician surveys confirm that non-financial incentives (such as stickers, risk disks, pens or candy) are not effective [2]. Three trials reported in this review used a pen or pencil as an incentive. Two of those studies found no effect. One Australian study compared four different methods of recruitment rather than using a control group [10], while another by Clark et al. [11] surveyed US obstetricians rather than family physicians. In line with our study, the third trial compared a pencil with a control group and found that the response rate was higher in the intervention group (32%) compared to the control group (17%) [4], whereas the authors also conducted a 2nd study comparing a pen versus a telephone reminder and did not find a significant effect. Recently a study amongst health care providers [12] showed that lottery tickets or non-financial conditional incentives did not lead to response rates above 60% but this included only three studies for each group. The authors call for improved reporting standards for health care provider surveys.

Despite the majority of general practices being computerised in Australia, no GPs completed the survey online. It is not yet well understood why physicians are less likely to complete online surveys [3]. It may be that we had zero responses because the GPs had to enter the URL link themselves. However, other studies confirm the extremely low response rates for online surveys among general practitioners [2,13,14]. For example, in an Australian study, GPs were invited via an email newsletter and found that less than 0.1% opted to complete the online survey [13] while 12.4% completed the postal survey. VanGeest et al. conducted a systematic review of 66 studies [2] amongst physicians and also found that postal and telephone surveys were more effective than web based surveys. Additionally, 81% of Australian GPs have nominated mails surveys as their preferred mode of administration [14].

### Implications for GP surveys

In conclusion, a small unconditional non-financial incentive, in the form of a pen, may improve response rates for GPs by an important margin.

### Implications for GP recruitment research

Further study is required to focus on the efficacy of non-financial incentives in the GP population, given the rather limited and somewhat conflicting results in this area to date. The acceptability and uptake of electronic communication is rapidly evolving and the preference for paper-based surveys may change. Recruitment of GPs for primary care research will remain a challenge and the research community will need to continually update the evidence for participant recruitment. We recommend that researchers who survey GPs build in a randomised controlled trial to test different recruitment methods in their research planning and budget to advance this field.

### Ethics approval

The study was approved by the University of Sydney Human Research Ethics Committee (ID 14112).

## Competing interests

The authors declare that they have no competing interests.

## Authors’ contributions

SWP designed the study, collected data, analysed and drafted the manuscript. VH collected data, and participated in study design. DE participated in the design of the study and gave advice. All authors read and approved the final manuscript.

## Pre-publication history

The pre-publication history for this paper can be accessed here:

http://www.biomedcentral.com/1471-2296/14/108/prepub
